# P03-017 - Health related quality of life in adult with HRFS

**DOI:** 10.1186/1546-0096-11-S1-A215

**Published:** 2013-11-08

**Authors:** HJ Lachmann, N Stewart, T Lane, DM Rowczenio, PN Hawkins

**Affiliations:** 1 University College London Medical School, London, UK; 2 National Amyloidosis Centre, University College London Medical School, London, UK

## Introduction

Hereditary recurrent fever syndromes (HRFS) can have a profound impact on health related quality of life (HRQoL) but there have been relative few prospective studies.

## Objectives

To assess HRQoL in patients aged over 17 years with confirmed CAPS, TRAPS and MKD and to assess the effect of treatment with biologic therapies.

## Methods

Patients completed the 36-item Short Form Health Survey (SF-36). Data were collected on untreated patients and those on long term treatment with biologics. SF-36 measures the impact of disease on overall quality of life. Results are expressed as physical-component summary score (PCS) and a mental-component summary score (MCS). Scores range from 0 at worst to 100 at best, with values greater than 50 indicating a better HRQoL than that of a normal adult in the United States.

## Results

In CAPS pre treatment HRQoL scores were available on 18 patients, 11 female, median age 43.6 years (IQ 27.9-47.1). The most affected domains were bodily pain, mean score 44, general health 32.5 and vitality 40.3. After treatment with anti IL-1 agents HRQoL was available on 31 patients, 13 female, median age 40.1 years (IQ 27.7:47.8). There was an improvement of more than 20 across all domains except role emotional which improved by 11 and scored above the US average on all domains. There was no significance difference in HRQoL in the 26 patients on long term canakinumab and the 8 on anakinra (one patient provided data on both agents). Data were available on 7 cases of TRAPS who have never received biologics (5 females, median age 443yrs (IQ: 24:44)) and 13 prior to biologics (10 females, median age 39 yrs (IQ 18.5:37)). HRQoL in patients managed with episodic steroids was near normal across all domains, mean PCS 49.7, MCS 52.6. In patients whose disease was severe enough to require biologics pretreatment scores were significantly lower, mean PCS 38.7 and MCS 43.0. In 19 TRAPS patients on biologics (13 female, median age 42.6 yrs (IQ 28:50), 3 with AA amyloidosis) HRQoL was significantly better across all domains, mean PCS 49.1 and MCS 51 than prebiologics but no different to those adequately managed with steroids. Of 6 patients with MKD (1 female, median age 28.1 yrs (IQ 24.4:31.8)) all had severe disease (5 on biologics , 4 refractory to etanercept, 3 refractory to anakinra, 1 refractory to tocilizumab, 1 with AA amyloidosis). Quality of life on no treatment in 2 cases and ineffective biologics in the others was poor, particularly general health (mean 22.8) and social function (mean 22.5), mean PCS 36.1, MCS 28.0. With effective treatment HRQoL (anakinra 1, canakinumab 1, tocilizumab 2) improved significantly across all domains in 4 assessed patients, mean PCS 48.5, MCS 46.8.

## Conclusion

HRQoL improves with effective long term treatment. Mean scores were worst in untreated MKD. Self reported improvement was most marked in CAPS c who reported sustained high scores on anti IL-1 agents. There was also improvement in patients with TRAPS and MKD and in treated patients with both diseases HRQoL was almost the US norm. HRQoL data aids holistic assessment of disease activity; the improvements reported here mirror changes in clinical disease activity and inflammatory markers on treatment and help justify the cost and potential long-term risks of biologic therapies.

## Competing interests

None Declared.

